# Acute Gastrointestinal Bleeding: An Update and a Practical Diagnostic Approach

**DOI:** 10.3390/diagnostics16060860

**Published:** 2026-03-13

**Authors:** Elio Antonucci, Ilaria Zanichelli, Alessandro Rimondi

**Affiliations:** 1 Intermediate Care Unit, Emergency and Intensive Care Medicine Department, Ospedale Guglielmo da Saliceto, 29121 Piacenza, Italy; 2 Department of Emergency Medicine, ASST Grande Ospedale Metropolitano Niguarda, 20162 Milan, Italy; 3 Interventional Digestive Endoscopy, ASST Spedali Civili di Brescia, 25123 Brescia, Italy

**Keywords:** acute gastrointestinal bleeding, diagnostics, endoscopy, critically ill patients

## Abstract

Acute gastrointestinal bleeding (GIB) is one of the most common and dangerous condition in patients admitted in Emergency Departments. The incidence and the mortality of acute GIB remain significant, although some positive trends were observed in recent years. Initial evaluation of GIB needs an accurate assessment of the medical history and the clinical presentation. Physicians should pay attention about the presence of hemorrhagic shock that usually requires urgent diagnosis and treatment. Only a prompt diagnostic approach can identify the source of bleeding and improve the outcomes in acute GIB patients. Risk stratification and time of endoscopy are fundamental issues in the management of upper and lower GIB. Small bowel capsule enteroscopy (SBCE) and device-assisted enteroscopy (DAE) are the basic approaches to suspected small bowel bleeding. Machine Learning Prognostic Models have been proposed, such as alternative prognostic tools in GIB, but they are currently recommended only to identify low-risk outpatients.

## 1. Introduction

Acute gastrointestinal bleeding (GIB) is a common and potentially life-threatening condition frequently affecting a very heterogeneous cohort of patients (i.e., elderly, cirrhotic, and otherwise healthy individuals).

The epidemiology of GIB is changing and it is crucial to understand emerging trends across different populations. The severity and timing of bleeding can range from mild to severe and from chronic to acute, respectively, often requiring immediate medical attention and intervention [1].

In particular, when a patient presents with acute GIB, a prompt assessment and an adequate resuscitation should be mandatory. A correct diagnostic approach to identify the exact source of bleeding is a cornerstone in GIB management and several novel techniques are available. In this article we provide a narrative review of the complex GIB field analyzing some different topics such as definition, epidemiology, risk factors, current diagnostic approach to UGIB, LGIB and SBB, and future perspectives in this field.

## 2. Review Methodology

We performed a narrative review, analyzing the current scientific literature on GIB. We searched for keywords such as “acute”, “diagnosis” and “gastrointestinal bleeding” on Pubmed, Scopus and Google Scholar and including articles published in the last 25 years. We considered only selected publications, in particular systematic reviews, meta-analyses and current guidelines. EA and IZ analyzed the articles focusing on epidemiology, initial diagnostics and UGIB issues; AR analyzed the articles about current GIB definition, LGIB and SBB.

## 3. Definition of Upper and Lower Gastrointestinal Bleeding

When approaching a GIB, choosing the correct approach and procedure is pivotal to ensure a correct identification of the source and its treatment. Conventionally and historically, GIB has been classified as “upper” (UGIB) when the source of bleeding is found from the mouth until the ligament of Treitz, therefore until the fourth duodenal section [2,3,4]. This definition reflects the practical endoscopic boundary, since the ligament of Treitz can be accessed with a standard gastroscope or a pediatric colonoscope, which are commonly available in most endoscopy units. Conversely, LGIB has been historically defined as any bleeding that originates distal to the ligament of Treitz, although, with the advent of enteroscopy techniques [5,6], this is considered an outdated definition. Hence, nowadays LGIB is the preferred definition for any bleeding that originates from the colon to the rectum (or from the ileo cecal valve) [7,8], whereas small bowel bleeding (SBB) should be the favored definition for bleeding involving any segment of the jejunum and the ileum [9].

## 4. Epidemiology, Trends and Risk Factors

GIB represents an important cause of Emergency Department visits in the United States (US), accounting for approximately 1,707,704 cases per year (2/3 UGIB, 1/3 LGIB). Severe GIB is also a leading cause of hospitalization and hospital readmission in US, placing a considerable financial strain on healthcare systems [10]. Epidemiologic data on SBB remain insufficient, although it is estimated to account for approximately 5% of all GIB cases worldwide [10]. Table 1 summarizes the most frequent causes of GIB. Different epidemiological trends have been reported in the literature for UGIB or LGIB.

### 4.1. UGIB: Epidemiological Trends and Risk Factors

Wuerth and Rockey performed a retrospective analysis of an adult population discharged with an ICD-9 principal diagnosis of UGIB showed an estimated incidence around 67 cases per 100,000 individuals, declining in the last 10 years [11]. The greatest reductions occurred for peptic ulcer disease (PUD) and gastritis, which decreased by 30 and 55%, respectively (*p* < 0.01). However, the same authors found an increase in neoplasms, Dieulafoy lesions, angiodysplasia, and esophagitis by 50, 33, 32 and 20%, respectively (*p* < 0.01) [11]. PUD remained the leading cause of acute UGIB, accounting for up to 67% of all cases [12], whereas esophageal variceal hemorrhage accounted for approximately 10–20% of all cases of UGIB with a hospitalization rate of approximately 2% throughout the study period. Regarding mortality, UGIB accounts for 4–10% of cases. The mortality for esophageal varices remained constant at 6–7%, whereas less frequent causes of mortality are usually caused by PUD, gastritis, esophagitis, and Mallory-Weiss tears. Rare causes of mortality also include tumor, Dieulafoy lesions, hemobilia, vascular malformations and/or aortoenteric fistula [13]. Marmo and Koch [14] performed a prospective analysis of 1020 consecutive patients endoscoped for UGIB and showed that 85% of deaths were associated with one or more major comorbidities. In particular, advanced age, severity of anemia at presentation, worsening health status and failure of endoscopic treatment were independent predictors of 30-day mortality (*p* < 0.001).

### 4.2. LGIB: Epidemiological Trends and Risk Factors

Data from some observational studies showed that LGIB occurs in approximately 36/100,000 people in the Western Countries annually and is associated with 2–9% mortality [15,16]. Common causes of acute LGIB, in descending order of prevalence, include diverticulosis, hemorrhoids, ischemic colitis, colorectal polyps or neoplasms, angiodysplasia, postpolypectomy bleeding, inflammatory bowel disease, and infectious colitis [15,16]. Rarer causes include stercoral ulceration, colorectal varices, radiation proctopathy, and nonsteroidal anti-inflammatory drug (NSAID)-induced colopathy [15,16] (see Table 1). Lanas et al. [17] analyzed time trends and impact of GI complications in 10 general hospitals between 1996 and 2005. The authors found a progressive change in the general incidence of GIB with clear increasing trends in LGIB. Overall mortality decreased, but the in-hospital case fatality remained constant. Common risk factors of severe LGIB were older age, a higher number of comorbidities and more recent years of diagnosis [17]. Furthermore, LGIB was more frequent in men than in women, which can be attributed to the higher prevalence of vascular diseases and diverticulosis in the male population.

## 5. Initial Evaluation of GIB

An accurate assessment of the medical history and the clinical presentation is therefore essential to guide further treatment. Melena (i.e., the emission of pitch-dark colored and foul-smelling liquid stool), coffee-ground vomiting, hematemesis (i.e., blood in the vomit) are classical signs of UGIB, whereas hematochezia (i.e., the emission of red blood or clots) and frank rectal bleeding suggest an LGIB. In cases of melena, some authors in the past suggested gastric tube placement in order to differentiate between upper and lower etiologies. Gastric fluid containing bile and no blood effectively makes UGIB less likely. However, the current recommendations [18] discourage gastric tube placement during acute UGIB as it may be associated with complications without a clear evidence of beneficial effects [18]. Some laboratory tests can add useful information. For example, a blood urea nitrogen-to-serum creatinine ratio greater than 30:1 suggests an upper gastrointestinal source of hemorrhage, likely related to heme absorption and metabolism.

## 6. Initial Diagnostics in Case of Hemorrhagic Shock

The initial GIB evaluation should include a correct stratification about the timing and severity of bleeding. Emergency physicians and intensivists need to discriminate an acute or chronic anemia due to GIB and identify a overt or occult hemorrhagic shock. Through history and initial physical examination, the clinicians should promptly treat a massive or frank GIB. The most relevant information about GIB is a prior history of GIB. Other relevant issues include the known presence of varices, portal hypertension, cirrhosis, alcohol use, diverticulitis, inflammatory bowel disease, or a history of colorectal cancer. A second step includes the evaluation of a possible hemorrhagic shock state. Shock is the clinical expression of circulatory failure that results in altered intracellular oxygen utilization [19]. Circulatory failure is a common finding in ICUs and Emergency Departments, accounting for about one third of critically ill patients [20]. Once the gastrointestinal source of bleeding is proven or highly suspected, a presumptive diagnosis of hemorrhagic shock needs a “three steps” approach to be confirmed. First, there is a systemic arterial hypotension (systolic arterial pressure < 90 mmHg or mean arterial pressure < 65 mmHg) usually associated with a compensatory tachycardia. It should pay close attention to chronic hypertensive patients that could show only a moderate hypotension in this setting [19]. Second, there are some clinical signs of tissue hypoperfusion such as cold or clammy skin, cyanosis, oligo-anuria (urine output of <0.5 mL per kilogram of body weight per hour) and altered mental state with confusion and disorientation [19]. Third, abnormal metabolism of intracellular oxygen induces variable grades of hyperlactatemia (plasmatic lactate concentrations > 2 mmol/L). A second level of evaluation may be required in a patient with proven hemorrhagic shock, including hemodynamic monitoring, to ensure accurate circulatory assessment and management. Hemorrhagic shock is a circulatory failure usually characterized by low cardiac output (due to hypovolemia), compensatory tachycardia, peripheral vasoconstriction and hyperlactatemia (inadequate oxygen transport). The diagnosis can be completed by a point-of-care echocardiographic (PoCUS) evaluation, which includes assessment for pericardial effusion, measurement of left and right ventricular size and function in order to estimate stroke volume and indirect signs of pulmonary hypertension [19]. Blood transfusions and hemodynamic stabilization should be managed simultaneously with a prompt diagnostic approach (CT scan, endoscopy, angiography), which often represents a cornerstone of GIB treatment.

## 7. Current Diagnostic Approach of UGIB

### 7.1. Risk Stratification

Several validated risk assessment scores are available for the prognostication of patients with UGIB and to ensure an appropriate patient management [21,22]. These can be divided into pre-endoscopic scoring tools (e.g., pre-endoscopic Rockall AIMS65, and Glasgow Blatchford scores) and post-endoscopic scoring tools (e.g., full Rockall score, Cedars Sinai, and Progetto Nazionale Emorragia Digestiva). In particular, pre-endoscopic scores allow an early stratification of patients into low- and high-risk groups, thus guiding appropriate diagnostic and therapeutic pathways and allocation to the correct level of care (general ward or intensive care unit [ICU]), while post-endoscopic scores require an endoscopic assessment to complete the full score, restricting their use in the initial management of UGIB, before endoscopy is performed [23,24]. The Glasgow Blatchford Score (GBS) is based only on initial clinical and biochemical parameters in the Emergency Department, including blood urea, hemoglobin level, systolic blood pressure, pulse rate, presence of melena, presence of syncope, and history of hepatic disease and heart failure, and it is used to stratify UGIB risk. It has been validated to identify patients that will require in-hospital management [25]. In fact, a low GBS (< or =1) has shown a high sensitivity in identifying patients with a very low-risk of needing in-hospital treatment and that can be safely discharged from hospital with an outpatient diagnostic follow-up [23]; conversely, a GBS higher than 2 is associated with higher probability of requiring an endoscopic hemostatic intervention and blood transfusions [26]. The clinical or pre-endoscopic Rockall score (pRS) is calculated using the anamnestic and clinical data of the patient (age, signs of shock and comorbidities) and has the aim of predicting pre-endoscopic mortality, while the full Rockall score (RS) also includes the post-endoscopic component (etiology of bleeding and presence of active bleeding) and is useful to calculate overall mortality, but it has proven to be poor in predicting the risk of rebleeding and only moderately accurate in predicting the need for endoscopy and blood transfusion [27,28]. AIMS65 score is a simple pre-endoscopy score that considers five parameters with equal weight, albumin, international normalized ratio (INR), mental status, systolic blood pressure and age > 65 years (AIMS65) and it can be easily calculated bedside [29]. Although easily applicable in the Emergency Department, it has shown accuracy in predicting in-hospital mortality, but it is significantly inferior to GBS in prognosticating the requirement of blood transfusion, the rebleeding risk and the stratification of low-risk patients who can be safely discharged from hospital [30]. For all these reasons, the GBS is the most accurate tool to stratify UGIB to this day.

### 7.2. Diagnostic Tools and Time of Endoscopy

In a case of suspected/proven UGIB, the diagnostic approach relies on esophagogas-troduodenoscopy (EGD), computed tomography (CT) angiography (angioCT) and angiography [1]. EGD allows the visualization of the upper GI tract from the mouth to the proximal duodenum and the application of endoscopic therapy to achieve hemostasis at visible bleeding sites. AngioCT is a non-invasive technique that allows us to identify arterial leakages with high output bleeding but needs a separate procedure to perform hemostasis—namely angiography. In this sense, standard angiography allows confirmation of the bleeding source and to real-time treatment through an embolization procedure or intra-arterial vasopressin infusion [1,31]. Endoscopy is defined as “emergent” if performed within 6 h following the presentation, as “urgent” if performed within 12 h, and as “early” if performed within 24 h. Early endoscopy, especially when performed after an adequate resuscitation and optimization of comorbidities, has shown the best outcomes, including lower in-hospital mortality, fewer procedures, shorter hospital stay, and lower total hospital costs [21,22]. In fact, performing an endoscopy in an emergency setting may be a high-risk procedure for several factors, such as inadequate resuscitation, procedures performed during night or weekend shifts with limited resources, or the presence of a single endoscopist, potentially leading to lower-quality evaluation, suboptimal hemostasis, and poorer outcomes [32,33]. The current practice guidelines of the European Society of Gastrointestinal Endoscopy (ESGE) [3] recommend that patients with nonvariceal UGIB should receive upper early endoscopy (within 24 h from presentation), only after adequate resuscitation, and do not recommend urgent (within 12 h) or emergent (within 6 h) endoscopy, as no clear improvement in outcomes has been demonstrated. Otherwise, it is important to underline that suspected variceal upper GIB has a different management: ESGE guidelines suggest an endoscopic evaluation and treatment within 12 h of patient presentation, always after hemodynamic resuscitation [34].

## 8. Current Diagnostic Approach of LGIB

Since the advent of therapeutic endoscopy, the paradigm of treatment of LGIB has shifted from surgery to endoscopy. The key prerequisites to deliver a safe and effective endoscopic treatment of LGIB are:

- Patient stabilization;
- Bowel preparation.

Stabilizing the patient is essential in LGIB as colonoscopy requires bowel preparation. There are many reasons to administer bowel prep in LGIB.

Colonoscopy is a stressful procedure and the risk of complications (both direct and indirectly related to colonoscopy) rises when hemostatic therapy is delivered [35]. It is not advisable to intervene in an unprepared colon, even when the source of bleeding is distal, as there is a risk of bowel explosion that is more frequent when the bowel preparation is absent or partial (i.e., enemas) [36].

After hemodynamic stabilization and resuscitation, the diagnostic modality of choice is colonoscopy.

### Timing of Endoscopy

Historically, 24 h has been considered as the correct time frame to intervene in LGIB (early colonoscopy), although recent studies have questioned the utility of this approach. Although early colonoscopy is associated with an increased rate of source and stigmata of bleeding identification, its effectiveness is somehow limited by an increased risk of rebleeding and a lack of improvement in the mortality and in the need for interventional radiology and surgery rates when compared with elective (24–48 h) and late (48–120 h) colonoscopy [37,38]. In this sense, the Oakland score is a validated tool that helps allocate patients to urgent or outpatient management. Its parameters include age, gender, hemodynamic variables, hemoglobin, and comorbidities, and a low cutoff (≤8) indicates a low-risk subgroup that might be safely managed without hospital admission [39,40,41].

**Figure 1:**
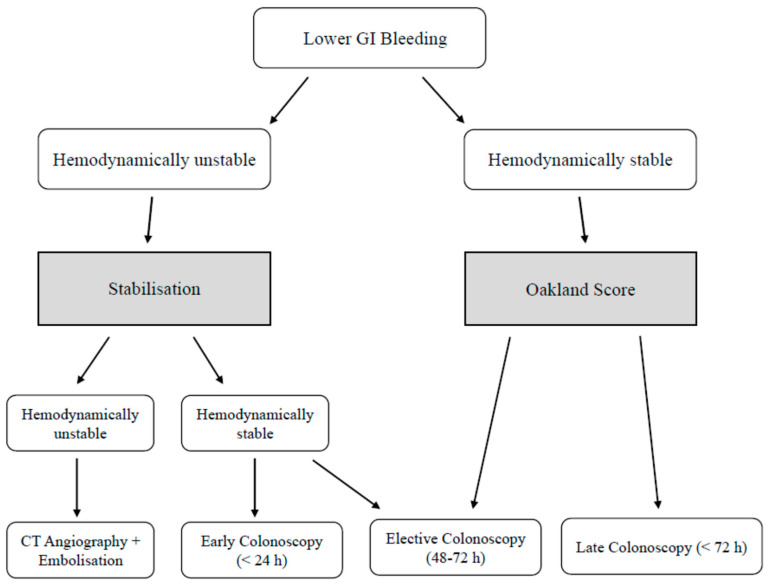
Algorithm of LGIB Management.

Instead, arterial LGIB requires a different approach. In this case, an hemodynamically unstable patient with a positive shock index usually necessitates an urgent CT angiography (CTA) to locate an active bleeding (often at rates ≥ 0.3–0.5 mL/min) and provide further therapy [7,41]. CT guided embolization has a high success rate but it is counterbalanced by an increased risk of bowel ischemia and need for surgery [42] (See Figure 1).

In few selected cases of refractory bleeding, surgery is still an option. In a study based on USA data, surgical treatment for LGIB was carried on 1.7% of the patients, usually hemodynamically unstable or with previous embolization attempt [43]. The timing of the surgical intervention is not clear.

## 9. Small Bowel Bleeding

Small bowel bleeding (SBB), as already mentioned, is defined as a bleeding that occurs from the ligament of Treitz to the ileo-cecal valve [9]. It accounts for roughly 5% of all the gastrointestinal bleeding, although data regarding its prevalence are outdated and scarce overall [44]. In this sense, updated studies assessing the magnitude of this phenomenon are needed.

The advent of small bowel capsule enteroscopy (SBCE) and device-assisted enteroscopy (DAE) in the early 2000s radically changed the approach of SBB that used to be reserved mostly for surgery or rudimentary push enteroscopy (i.e., an upper GI endoscopic examination performed with a longer colonoscope) [5,6].

Nowadays, these two methods are the basic approaches to suspected SBB (SSBB)—when a patient has already received bi-directional endoscopy (EGDS and colonoscopy) for a suspected GI loss [9].

Usually, SBCE is reserved as the first diagnostic step for SSBB. That is due to the safety profile of this examination and the improved diagnostic yield of DAE when SBCE is performed beforehand [45]. General recommendations when performing an SBCE include careful assessment of past medical history and current symptoms, as SBCE is associated with a 2% risk of capsule retention that grows up to 10% in case of inflammatory bowel disease. Caution is also advised in case of dysphagia and previous history of bowel resection or radiotherapy [46].

However, there is no agreement between guidelines to propose SBCE as the first diagnostic step. The Japanese guidelines [47,48] advocate a more widespread use of CT scan as the preliminary test for SBB. This is especially suggested for younger patients who have higher prevalence of non-vascular pathologies (i.e., cancers) and possible extra luminal masses, or for those patients who are hemodynamically unstable [49].

It is not well demonstrated that the diagnostic yield of diagnostic procedures performed on the small bowel is time-dependent with earlier procedures associated with increased findings [50]. The proposed timing of these investigations by the updated European guidelines is 48 h from the bleeding episode [9]. However, difficulties in keeping up to this timing schedule have been reported. In a European questionnaire proposed to 91 experts, only 31.3% stated that the timing of the SBCE was correct for SSBB [51]. The evidence, though, is far from being consistent, with recent studies reporting optimal diagnostic yields of SBCE in SSBB even 14 days after clinical presentation [52].

**Figure 2:**
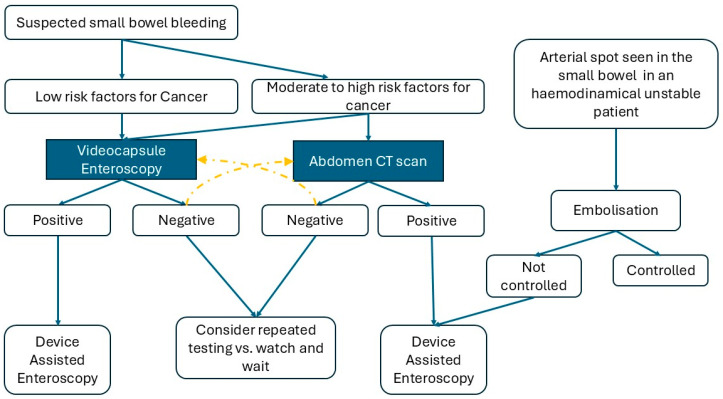
Algorithm for initial SSBB Management. Yellow arrow: Consider as alternative approach. The colors of the boxes emphasize the different approaches represented in the figure.

Despite having a diagnostic purpose as well, DAE is usually reserved as a therapeutic procedure since it can provide direct hemostasis, and can locate and acquire biopsies and mark lesions [9,48] (See Figure 2).

## 10. Future Perspectives

Early stratification of bleeding severity will increasingly become a key element in the management of GIB patients. In particular, a correct stratification should help the clinicians distinguish between patients who require admission in ICU or general ward and those who can be managed as outpatients. There are tools such as the Acute Physiology and Chronic Health Evaluation (APACHE) III score or SAPS II (Simplified Acute Physiology Score II) that help to estimate the mortality of patients already admitted to the ICU [53]. APACHE III score is a prognostic tool calculated by adding points from three components: an Acute Physiology score, an Age score, and a Chronic Health Evaluation score (e.g., history of cirrhosis, metastatic cancer, or immunosuppression). The total score ranges from 0 to 299, with a higher score indicating a greater risk of hospital death. This score is calculated using 17 physiological measurements, age, and chronic health conditions, with data from the first 24 h of ICU admission being used for initial assessment [53]. SAPS II is a scoring system used in ICUs to assess the severity of illness for patients aged 15 or older. It uses a 24 h period post-admission to collect data on 12 physiological variables, age, chronic health status, and the type of admission to generate a score between 0 and 163. This score is then used to predict a patient’s risk of death, and it is primarily used for comparing patient groups, not for determining the outcome for an individual patient. However, these scores have shown important limitations and are mainly applicable in ICU settings. For these reasons, alternative tools with improved accuracy have been developed, including Machine Learning (ML) prognostic models. Machine Learning Prognostic Models are frameworks that use algorithms to predict future health outcomes based on patient data, offering a more flexible and accurate alternative to traditional methods [54]. These models can predict outcomes like survival time or disease progression and are applied across various specialties, such as oncology, immunology, and critical care medicine. ML models can analyze a large set of patient data, including factors such as genetics, medical history, and responses to treatments. ML usually identify complex patterns and relationships within the data that are often too intricate for traditional statistical models to capture. Based on these patterns, the model generates an individualized prediction for a future outcome, such as the probability of survival or disease recurrence [54]. Although ML offer significant advantages over traditional approaches, they are currently recommended mainly to identify low-risk patients who may be managed as outpatients. Recently, there has been a growing interest in less invasive screening tools such as blood sensing capsules when the clinical presentation of GIB is unclear. Two devices, consisting of small dimension capsules with an optical sensor, have been recently tested in small prospective clinical trials. Both devices have shown potential in identifying appropriate candidates for endoscopic treatment in case of unclear GIB [55,56]. However, there is still uncertainty regarding which population will benefit from the application of these devices, their cost-effectiveness, and their safety and retention rate when administered in large numbers of patients.

Conversely, there has been an ongoing debate about whether pan enteric capsule endoscopy can be potentially used as a tool to identify the source of bleeding as a first screening tool. Although feasible, it has never been adopted in clinical practice due to the time required to administer the capsule, the time to download and analyze the recorded material, and the different diagnostic yield in the GI tract (e.g., esophagus and colon) [57,58,59]. However, recent developments in capsule endoscopy technology (i.e., real-time reading, automated AI-driven reading) could potentially represent a future game-changer.

## 11. Conclusions

Acute GIB remains a significant clinical problem especially in patients admitted to Emergency Departments and ICUs. Morbidity and mortality continue to be high, particularly among patients with risk factors such as cirrhosis, elderly age, mechanical ventilation, and co-existing coagulopathy or kidney injury. The management of UGIB, LGIB and SBB necessitates a comprehensive, multidisciplinary approach involving risk stratification, an adequate diagnostic approach and timely resuscitation. Early EGD should be performed in cases of nonvariceal UGIB whereas suspected variceal bleeding needs an endoscopic evaluation within the first 12 h of hospital admission. The Oakland score end shock index could help discriminate the need of CT angiography or colonoscopy in a case of LGIB. Clinically significant SBB could be initially approached with a CT scan and secondly with a SBCE. Although recent developments have reduced the overall incidence of GIB, several aspects still require further improvement, particularly risk stratification. Future research should focus on alternative prophylactic strategies, early GIB detection, new therapeutic options and optimization of the prognostic model.

## Figures and Tables

**Table 1:** Common causes of gastrointestinal bleeding (GIB)—Abbreviations: IBD, inflammatory bowel diseases; NSAID, non-steroid anti-inflammatory drugs.

Upper GIB	Lower GIB
Variceal Hemorrhage	Diverticulosis
Peptic Ulcer Disease	Hemorrhoids
Esophagitis	Ischemic colitis
Gastritis	Colorectal polyps or neoplasm
Mallory-Weiss Tears	Angiodysplasia
Dieulafoy lesions	Postpolypectomy bleeding
Tumor	IBD
Vascular Malformation	Infectious colitis
Hemobilia	Stercoral ulceration
Aortoenteric fistula	Colorectal varices
	Radiation proctopathy
	NSAID-induced colopathy

## Data Availability

Not applicable. No new data were created or analyzed in this study.
